# Effects of thermocycling on color stability and mechanical properties of universal resin composites: an in vitro comparison

**DOI:** 10.1186/s12903-026-08987-z

**Published:** 2026-07-23

**Authors:** Alana Pinto Caroso Souza, Wilton Lima dos Santos, Camila Ribeiro Silva, Anderson Gomes Forte, Fernanda Midori Tsuzuki-Gorham, Américo Bortolazzo Correr

**Affiliations:** Dental Materials Division, Department of Restorative Dentistry, Piracicaba Dental School, State University of Campinas (FOP/UNICAMP), Av. Limeira, 901, Piracicaba, São Paulo 13414-903 Brazil

**Keywords:** Dental materials, Universal composite resins, Color stability, Mechanical properties

## Abstract

**Objectives:**

To evaluate the effect of thermocycling on the optical and mechanical properties of universal resin composites, with emphasis on color stability, Knoop microhardness, flexural strength, flexural modulus, and filler characteristics.

**Methods:**

Four composite resins: Filtek Z250 XT (FZ), Vittra APS Unique (VT), Spectra Smart (SS), and Harmonize (HZ) were evaluated (n = 10 per group). Specimens were subjected to thermocycling (10,000 cycles; 5–55 °C). Color and Knoop microhardness were measured before and after aging (repeated measures), while flexural strength and modulus were determined using independent specimens. Color differences were calculated using the CIEDE2000 formula. SEM/EDS analyses were performed for filler characterization. Statistical significance was set at α = 0.05.

**Results:**

Thermocycling significantly affected all evaluated properties (p < 0.05), with a material-dependent response. All materials exhibited ΔE₀₀ values above clinically acceptable thresholds after aging. SS showed the lowest color variation, while VT presented the highest values. In terms of mechanical behavior, FZ and HZ exhibited greater reductions in flexural strength (> 50%) and microhardness (≈ 30–45%), whereas SS demonstrated the highest stability and VT showed intermediate performance. Additionally, FZ and HZ presented flexural strength values below 80 MPa after thermocycling.

**Conclusions:**

Thermocycling resulted in clinically relevant color changes in all evaluated universal resin composites, exceeding perceptibility and acceptability thresholds. Additionally, mechanical properties were adversely affected in a material-dependent manner, with reductions in flexural strength and microhardness suggesting decreased structural stability after aging.

**Clinical Significance:**

Universal resin composites may exhibit clinically relevant color changes and significant mechanical degradation after aging. Clinicians should consider that materials with greater reductions in flexural strength and microhardness may present reduced durability over time.

## Introduction

Over the past decades, restorative dental materials, particularly resin composites, have undergone continuous development aimed at improving their mechanical performance, longevity, and aesthetic outcomes, thereby expanding their applicability across diverse clinical scenarios [1, 2]. These advances have been largely driven by modifications in filler technology, including particle reinforcement and size reduction, which have contributed to enhanced polishability and increased wear resistance [3–5]. In parallel, significant progress has been achieved in the organic matrix, especially through the development of novel monomer systems designed to reduce polymerization shrinkage stress and improve overall material stability [1, 6].

The selection of restorative materials, however, remains a critical step influenced by both environmental factors and operator-dependent variables [7, 8]. In response to the growing demand for simplified and predictable restorative protocols, universal resin composites, also referred to as single-shade materials, have been introduced to achieve broad shade matching through optical blending [7, 9]. These materials are intended to reduce the complexity of shade selection by harmonizing with the surrounding tooth structure, regardless of the initial shade chosen [6, 10]. Achieving and maintaining accurate color matching between resin composites and natural dental tissues has long represented a significant clinical challenge [7, 9].

Traditional composite systems rely on a wide range of shades and varying degrees of opacity and translucency, making shade selection highly technique-sensitive and dependent on the clinician’s experience and subjective judgment. In many clinical situations, this complexity leads to a trial-and-error approach, potentially resulting in unsatisfactory aesthetic outcomes and the need for restoration replacement, thereby increasing clinical time and costs [11, 12].

In this context, the color generation mechanism employed by non-pigmented universal resin composites represents a major conceptual shift [13]. Unlike conventional materials, in which color is predominantly determined by selective light absorption through pigments, these materials rely on selective light reflection produced by precisely engineered nanostructures [14]. This phenomenon, known as structural color, can be achieved through uniformly sized spherical nanoparticles with diameters smaller than the wavelength of visible light, enabling natural color blending with hard dental tissues [15, 16]. Structural coloration arises from fundamental optical phenomena such as light scattering, interference, and diffraction, and is generally considered more stable and less susceptible to discoloration than pigment-based systems [17].

Despite these advances, resin composites continue to exhibit intrinsic limitations related to their polymeric nature, including degradation, polymerization shrinkage, and susceptibility to staining over time [18]. When exposed to the oral environment, composites may undergo progressive changes in both mechanical and optical properties due to water sorption, chemical degradation, enzymatic activity, pH fluctuations, and thermal stresses [19, 20]. These factors collectively intensify material aging and may compromise the long-term success of restorative procedures.

Thermocycling is a well-established in vitro aging method used to simulate the thermal fluctuations and humidity conditions of the oral environment, promoting the degradation of resin-based restorative materials over time [21, 22]. In this context, color analysis is essential to assess aesthetic stability, since aging can alter luminosity and chromatic coordinates due to changes in refractive index matching and matrix degradation [23]. Microhardness provides an indirect indication of the integrity of the polymer network and resistance to water-induced softening, while flexural strength and flexural modulus assess load-bearing capacity and stiffness, reflecting structural durability [24, 25]. Analysis of filler particles also allows for the characterization of morphology and chemical composition, helping to explain differences in optical and mechanical behavior between materials subjected to thermocycling [26].

Despite the rapid development and growing clinical adoption of universal resin composites, most available studies have primarily focused on their immediate optical behavior and color adjustment ability under non-aged conditions. However, limited evidence exists regarding how artificial aging processes, such as thermocycling, influence the interplay between their optical performance and mechanical integrity. Furthermore, the extent to which structural color mechanisms, based on nanoscale light reflection rather than pigment absorption, remain stable after thermal and hydrolytic challenges is still not fully understood. This represents a critical knowledge gap, as both aesthetic stability and mechanical durability are essential for the long-term clinical success of these materials.

Therefore, the objective of the present study was to evaluate the effect of thermocycling on the optical, mechanical, and microstructural properties of universal resin composites with single-shade behavior. The experimental factors included the type of composite resin and the aging condition (before and after thermocycling), while non-aged specimens served as the experimental control. The evaluated parameters comprised color stability, microhardness, flexural strength, flexural modulus, and filler particle characteristics. The null hypotheses tested were: (1) thermocycling does not induce color alteration in universal resin composites; and (2) thermocycling does not affect the mechanical properties of universal resin composites. In this study, the term “universal resin composites” is used to describe materials designed to match multiple tooth shades, while “single-shade” refers to their optical behavior based on structural color and blending mechanisms.

## Materials and methods

### Experimental design

**Fig. 1:**
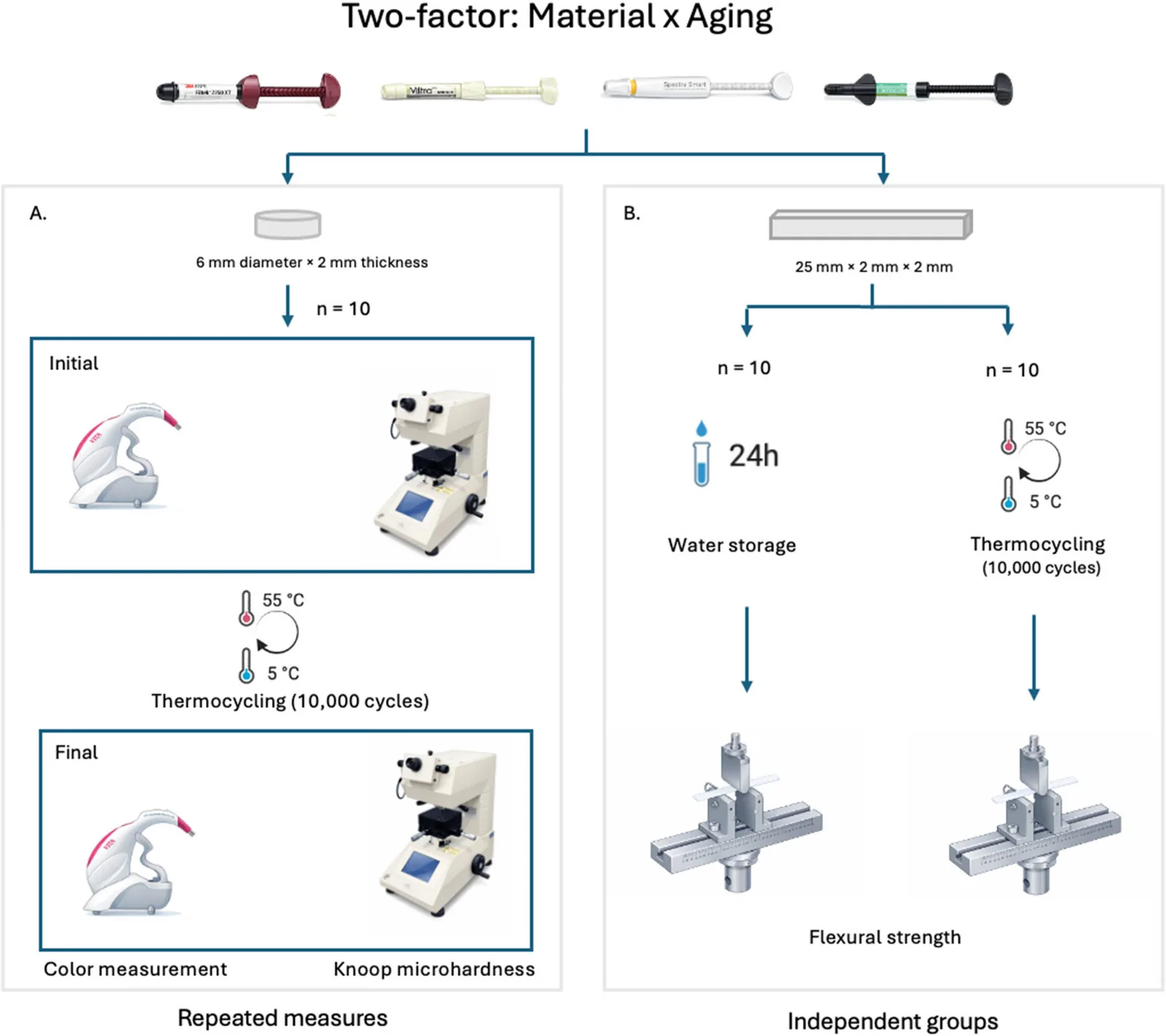
The experimental design consisted of a two-factor model (Material x Aging). For color and Knoop microhardness, the same specimens were evaluated before and after thermocycling (repeated measures design; *n* = 10 per material). For flexural strength, independent specimens were used (*n* = 10 per condition). Aging was performed by thermocycling (10,000 cycles, 5–55 °C, 15 s dwell time, transfer time 10 s), corresponding to approximately 12 months of clinical service

The experimental design was previously defined and schematically organized according to the nature of each test, as illustrated in Fig. 1. A mixed design combining repeated-measures and independent specimens was adopted. Color and microhardness analyses were performed using a repeated-measures approach, as these tests are non-destructive and allowed the same specimens to be evaluated before and after thermocycling, minimizing inter-specimen variability. In contrast, flexural strength testing was conducted using independent specimens for each condition, since this method is inherently destructive and results in specimen fracture. Therefore, separate specimens were required for baseline and aged conditions.

### Sample size

Sample size calculation was performed using G*Power 3.1 (Heinrich Heine University, Düsseldorf, Germany), considering a two-tailed test, α = 0.05, and statistical power of 80%. The effect size was set at f = 0.655, corresponding to a large effect size according to Cohen’s classification. This value was defined based on preliminary data obtained from a pilot study, in which three specimens per group were prepared and subjected to the same experimental conditions described in the present study, including thermocycling and evaluation of the primary outcomes (color change and mechanical properties). Based on these data, the minimum required sample size was calculated as 9 specimens per group; therefore, *n* = 10 was adopted for all experimental groups.

### Sample preparation

Four composite resins were used in this study: (FZ) Filtek Z250 XT; (VT) Vittra APS Unique, (SS) Spectra Smart and (HZ) Harmonize. The resin composites, manufacturer, composition, and lot are presented in Table 1. The specimens were made in an elastomeric mold (Express putty; 3 M ESPE, St. Paul, MN, USA) with 6.0 mm in diameter by 2.0 mm in thickness. The composite resins were inserted into the mold in a single increment of 2 mm, covered with a polyester strip and a glass plate. Then, they were light-cured for 40 s with an LED (VALO, Ultradent Products Inc., South Jordan, UT, USA). The composite resin discs were removed from the matrix and stored in distilled water at 37 °C for 24 h. After this period, the specimens were polished using silicon-carbide abrasive papers #400, #600 and #1200-grit (Norton; São Paulo, SP, Brazil) in a water-cooled automatic polisher (APL4; Arotec, Cotia, SP, Brazil).

**Table 1:** Composite resins used in the present study

Materials	Manufacturer	Type	Composition	Lot
Filtek Z250 XT (FZ)	3 M ESPE Dental Products, St. Paul, MN, USA	Nano-hybrid	Monomers: BIS-GMA, UDMA, Bis-EMA, PEGDMA and TEGDMA. Particles: Surface-modified zirconia/silica with a median particle size of approximately 3 microns or less. Non-agglomerated/non-aggregated 20 nm surface-modified silica particles. Concentration: The filler loading is 82% by weight (68% by volume).	2,100,800,342
Vittra APS Unique (VT)	FGM, Joinville-SC, Brasil	Nano-hybrid	Monomers: Mixture of methacrylate monomers; Particles: Boron-aluminium-silicate glass particles. Other components: Fhotoinitiator composition (APS), Camphorquinone, co-initiators, stabilizers and silane. Concentration: Inorganic filler content is 72% to 80% by weight (52% to 60% by volume), with average size filler particles of 0.02 to 0.7 microns	021020
Spectra Smart (SS)	Dentsply Sirona; Konstanz, Alemanha)	Nano-hybrid	Monomers: TCD-urethaneacrylate, UDMA, Methacrylate modified polysiloxane (organically modified ceramic); Dimethacrylate resins; Particles: Barium aluminum fluoride glass, Glass powder. Other components: Silica, Colloidal Hydrophobe, Dimethacrylate, Benzophenone III, EDAB, FluBlau Concentrate, Camphorquinone, BHT Butylated Hydroxytoluene, Yellow Iron Oxide, Red Iron Oxide, Black Iron Oxide and Titanium Dioxide.	364,506 L
Harmonize (HZ)	Kerr Dental, Orange, CA, USA	Nano-hybrid	Monomers: Bis-GMA, Bis-EMA, TEGDMA. Particles: Spherical silica and zirconia particles 5 to 400 nm formed from a molecular suspension in ART, barium glass.	7,897,660

The composition information provided in the Material Safety Data Sheet (MSDS) and the respective manufacturers' instructions

*BIS-GMA* Bisphenol A diglycidyl ether dimethacrylate, *UDMA *urethane dimethacrylate, *Bis-EMA* Bisphenol A polyethylene glycol diether dimethacrylate, *PEGDMA *poly (ethylene glycol) dimethacrylates, and *TEGDMA *tetraethylene glycol dimethacrylate

### Initial color measurement

After polishing, color was measured according to the CIE L*a*b* color scale relative to the standard illuminant D65 (daylight correlated with a temperature of 6500 K) over a white background using a spectrophotometer Vita Easyshade (Advance 4.0, Vita Zahnfabrik, Bad Sackingen, Germany). The resin specimens were properly dried with absorbent paper and 3 measurements were carried out in the center of the sample by the same operator.

The color coordinate “L*” is an achromatic coordinate and refers to the lightness ranging from black (0) to white (100). The coordinate “a*” is a chromatic coordinate that represents the green-red axis, in which negative values indicate green and positive values indicate red hue/chromas. The coordinate “b*” is also a chromatic coordinate that represents the blue-yellow axis, in which negative values indicate blue and positive values indicate yellow hue/chromas.

### Thermo-cycling

Resin specimens were submitted to a thermocycling challenge using a thermocycling machine (MSCT-3, Marcelo Nucci ME Instrument, São Carlos, SP, Brazil), programmed to perform 10,000 thermal cycles between 5 °C and 55 °C, with a dwell time of 15 s at each bath temperature and a transfer time of 10 s between baths. The immersion medium consisted of distilled water. This aging protocol was selected based on previously established laboratory procedures and has been suggested to correspond approximately to one year of clinical service, although such equivalence should be interpreted with caution due to the multifactorial nature of intraoral conditions [27, 28].

### Final color measurement

The differences in the values of L* (ΔL), a* (Δa), and b*(Δb) (where ΔL* is lightness, Δa* the differences in green-red, and Δb* the differences in the blue-yellow coordinates) were determined for each specimen, comparing the baseline values with the values obtained after thermocycling. The color difference was calculated using the CIEDE2000 formula:

$$\begin{aligned}\triangle\mathit{E00} = &[\left(\triangle\mathit{L}/\mathit{kL.SL}\right)\mathit{2} + \left(\triangle\mathit{C}/\mathit{kC.SC}\right)\mathit{2} + \left(\triangle\mathit{H}/\mathit{kH.SH}\right)\mathit{2}\\& + \mathit{RT}.\left(\triangle\mathit{C}/\mathit{kC.SC}\right).\left( \triangle\mathit{H}/\mathit{kH.SH}\right)]\mathit{0.5}\end{aligned}$$

Where, ∆L, ∆C and ∆H are the differences in lightness, chroma and hue, and RT is a function (the rotation function) that accounts for the interaction between chroma and hue differences in the blue region. The weighting functions, SL, SC, and SH are used to adjust the total color difference for variation in the location of the color difference pair in the L, a, and b coordinates. The parametric factors KL, KC, and KH, are correction terms for the experimental conditions, which were set to 1.

### Microhardness knoop (KHN)

The same disc-shaped specimens previously used for color evaluation were used for Knoop microhardness analysis before and after thermocycling (*n* = 10). Prior to testing, the specimens were polished on the irradiated surface using silicon carbide abrasive papers with decreasing grit sizes, followed by ultrasonic cleaning in distilled water to remove surface debris, and stored dry at 37 °C for 24 h to ensure stabilization.

Knoop microhardness measurements were performed using a microhardness tester (HMV-2000, Shimadzu, Tokyo, Japan). A static load of 10 gF was applied for a dwell time of 20 s, ensuring that the indentation depth did not exceed 10% of the specimen thickness. Three equidistant indentations were made on the surface of each specimen, with a minimum distance of 100 μm between indentations to avoid interference effects. The mean Knoop hardness number for each specimen was calculated and used for statistical analysis, as microhardness is considered an indirect indicator of polymer network integrity and resistance to softening associated with aging.

### Flexural strength (FS) and Flexural modulus (FM)

A three-point bending test was performed to obtain the FS (MPa) and E (GPa) of all RBCs using ISO 4049 protocols [26], using a universal testing machine (Instron—Model 4411, Norwood, MA, USA). Rectangular bars (2.0 × 2.0 × 25.0 mm) were produced in metal molds placed between two glass slides and light-curing using VALO (Ultradent Products Inc., South Jordan, UT, USA) for 20 s, with three overlapping exposures on each side, making six irradiations in total. Half of the bars of each group were stored in distilled water at 37 °C for 24 h (*n* = 10) and the other half taken to thermocycling (*n* = 10).

Specimens were tested at 1 mm/min cross-head speed with 20 mm distance between supports until fracture, The equation used to calculate FS (MPa) was:

$$\begin{aligned}\mathit{FS} = \mathit{3Fl} / \mathit{2bh}^2\end{aligned}$$

Where: F maximum fracture load (N), l distance between the supporting rollers (20 mm); b specimen width (~ 2 mm); and h specimen height (~ 2 mm).

While E (GPa) were calculated from the following formula:

$$\begin{aligned}\mathit{E}= \mathit{FI3}/\mathit{4dbh3}\end{aligned}$$

Where: I distance between the supporting rollers (20 mm), b specimen width (~ 2 mm), h specimen height (~ 2 mm), F maximum fracture load (N), and d deflection of the bar (in the elastic phase).

### Scanning electron microscopy (SEM)/Energy dispersive x-ray (EDX)

The filler morphology was determined using scanning electron microscopy (SEM). Unpolymerized monomers were removed by a washing technique: 0.5 g of each material was dissolved in 7 mL of ethanol and centrifuged for 5 min. This process was repeated using acetone and chloroform to ensure complete removal of the organic matrix. The remaining fillers were suspended in 1 mL of acetone and dried at 37 °C for 24 h. After gold coating, the fillers were examined using SEM (JEOL JSM 5600 PV, Japan) at magnifications of 5000× and 10,000×, operating at 15 kV.

Particle size was quantitatively measured using ImageJ software (Wayne Rasband, NIH, USA), with calibration performed based on the scale bar of each micrograph. For each material, three representative images (*n* = 3) were analyzed, and at least 30 particles per image were measured to obtain a representative distribution. Mean particle size and standard deviation were calculated.

An energy-dispersive spectroscopy (EDS) analysis was performed to qualitatively and semi-quantitatively identify the chemical composition of the filler particles. The samples were carbon-coated using a sputtering process (Desk II with Carbon Accessory, Denton Vacuum, USA) and analyzed using an EDS system (VANTAGE, Noran Instruments, USA).

### Statistical analysis

Statistical analyses were performed using SigmaPlot 12.0 (Systat Software, CA, USA). Data normality was assessed using the Shapiro–Wilk test, and homogeneity of variances was evaluated using Levene’s test. Knoop microhardness (KHN) data were analyzed using two-way repeated measures ANOVA (inter-subject factor: material; intra-subject factor: thermocycling), followed by Tukey’s post hoc test. Flexural strength (FS) and flexural modulus (FM) were analyzed using two-way ANOVA, considering material and thermocycling as independent factors. Color parameters (Δa, Δb, and ΔE00) were analyzed using the Kruskal–Wallis test followed by Dunn’s post hoc test due to deviations from normality. The ΔL parameter was analyzed using one-way ANOVA followed by Tukey’s test.

The use of both parametric and non-parametric tests was based on the results of normality and homogeneity analyses to ensure appropriate statistical handling of the data. Effect sizes were expressed as partial eta squared (ηp²), and their magnitude was interpreted as small (0.01), medium (0.06), or large (≥ 0.14). Additionally, 95% confidence intervals (95% CI) were calculated and are reported for the main outcomes (ΔE00 and flexural strength) to improve the interpretation of data precision. The level of significance was set at 95% (α = 0.05).

## Results

### Color measurements

Shapiro–Wilk analysis indicated that Δa, Δb, and ΔE00 data did not fully meet normality assumptions (*p* < 0.05), whereas ΔL data showed normal distribution (*p* > 0.05). Levene’s test confirmed homogeneity of variances for ΔL (*p* = 0.312). Therefore, non-parametric analysis was applied for Δa, Δb, and ΔE00, while parametric analysis was used for ΔL. The results of color variation expressed by the CIELAB coordinates (L*, a*, and b*) are summarized in Table 2. For ΔL, SS and HZ exhibited the lowest variation, indicating greater stability in brightness, whereas VT showed significantly higher ΔL values (*p* < 0.001), suggesting increased lightness after aging.

For Δa, FZ, VT, and HZ showed negative values, indicating a shift toward green, while SS showed positive values, corresponding to a red shift (*p* < 0.001). Regarding Δb, all materials except VT showed increased values, indicating yellowing, whereas VT showed negative values (*p* < 0.001), indicating a shift toward blue. Significant differences among materials were observed for all color coordinates (*p* < 0.05).

**Table 2:** Mean ± standard deviation of color changes and percentage of variation for each CIELAB coordinate (L, a, and b*) after thermocycling

	Color measurement (L*, a* and b*)		
	ΔL	Δa	Δb
FZ	6.45 ± 2.35 b	-0.8 ± 0.25 b	3.1 ± 2.37 bc
VT	11.88 ± 2.44 a	-1.3 ± 1.07 b	-3.3 ± 1.03 c
SS	-0.02 ± 2.35 c	1.0 ± 0.72 a	5.7 ± 2.33 ab
HZ	0.85 ± 4.14 c	-0.6 ± 1.35 b	11.0 ± 5.84 a

* Different lowercase letters indicate significant differences between the materials (*p *< 0.05)

**Fig. 2:**
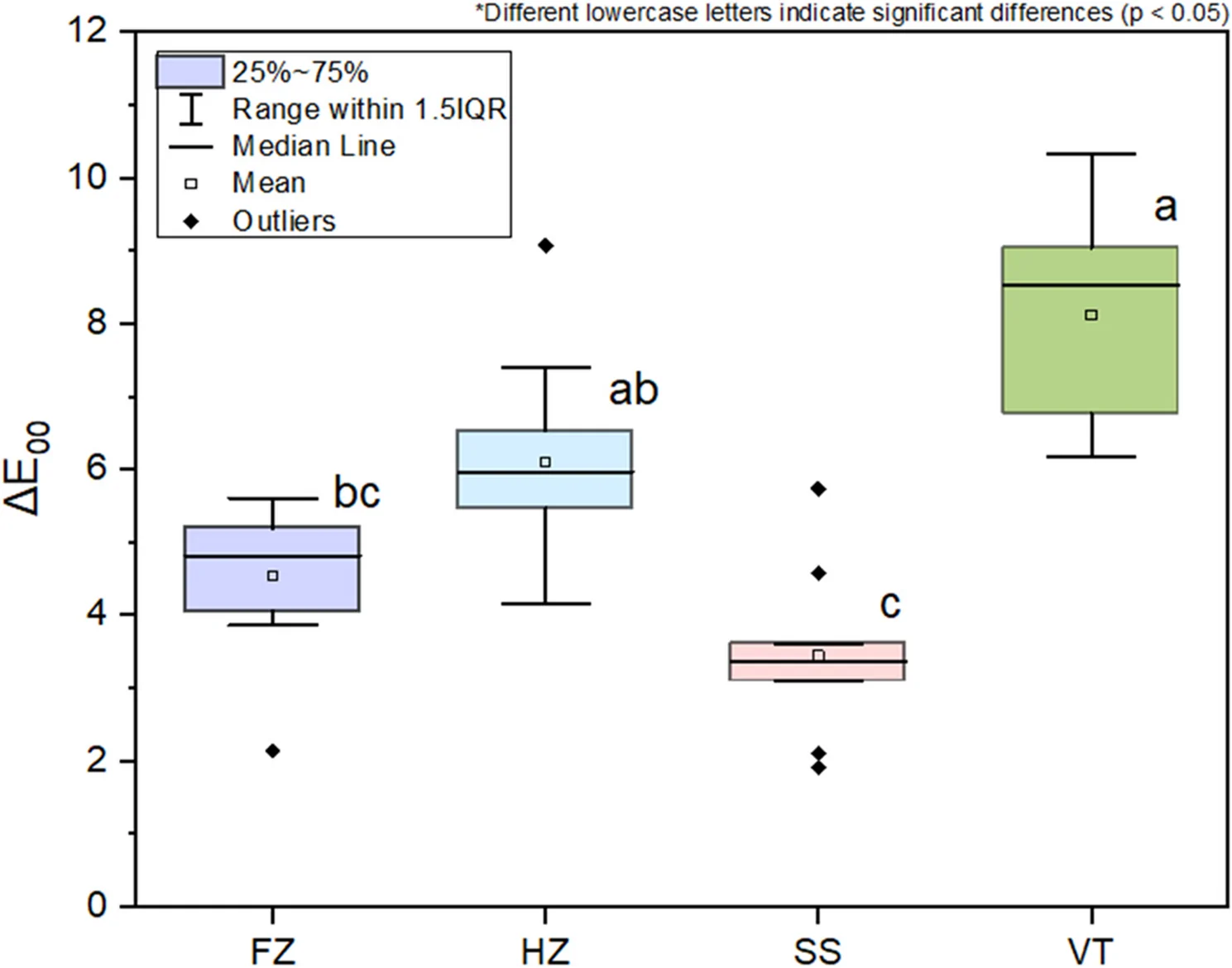
Boxplot of color change (ΔE₀₀) of the composite resins after thermocycling

The values obtained in the evaluation of the color parameters of the specimens (CIEDE2000) before and after thermocycling are described in Fig. 2. For ΔE00, significant differences were observed among materials (*p* < 0.001). SS showed the lowest color change (3.44 ± 1.10; 95% CI: 2.65–4.23), followed by FZ (5.46 ± 1.08; 95% CI: 4.69–6.23), HZ (6.30 ± 1.32; 95% CI: 5.36–7.24), and VT, which presented the highest values (8.11 ± 1.40; 95% CI: 7.11–9.11). From a clinical perspective, all materials exhibited perceptible color change after thermocycling, with VT presenting the most pronounced optical instability. All evaluated materials exhibited ΔE00 values above the clinically acceptable threshold, indicating that thermocycling resulted in clinically unacceptable color changes regardless of the material.

### Mechanical tests

#### Knoop microhardness (KHN)

**Fig. 3:**
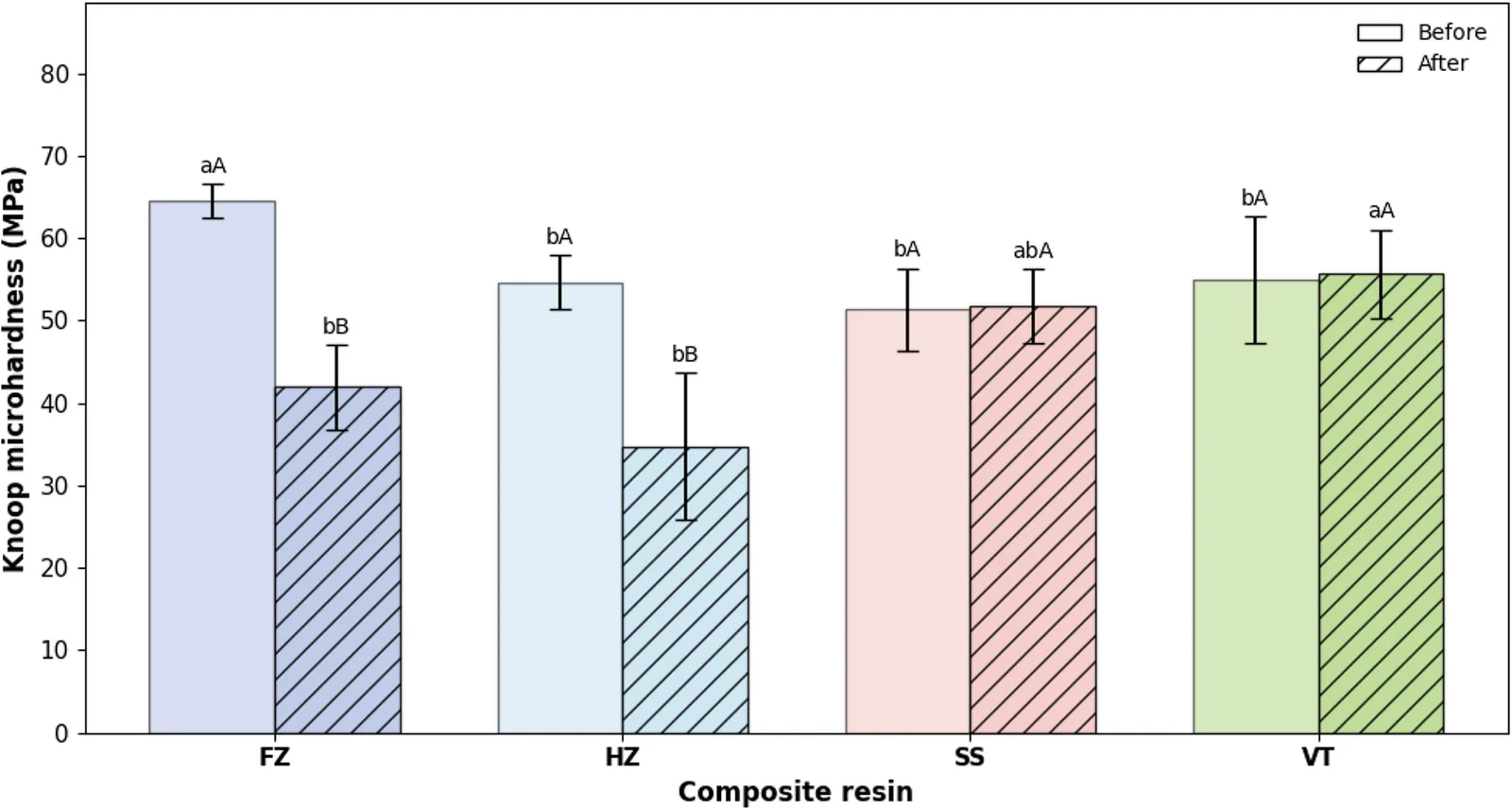
Mean ± standard deviation of Knoop microhardness (KHN) values before and after thermocycling. *Different capital letters indicate significant differences for thermocycling (before or after) from Tukey’s test (*p* < 0.05). **Different lowercase letters indicate significant differences between the materials from Tukey’s test (*p* < 0.05)

Shapiro–Wilk and Levene’s tests indicated normal distribution (*p* > 0.05) and homogeneity of variances (*p* = 0.274) for KHN data, supporting the use of parametric analysis. Two-way repeated measures ANOVA revealed significant effects of material (*p* < 0.001; ηp² = 0.44), thermocycling (*p* < 0.001; ηp² = 0.33), and their interaction (*p* < 0.001; ηp² = 0.29), indicating a large magnitude of effect. Post hoc analysis (Tukey) showed that thermocycling significantly reduced KHN values for FZ and HZ (*p* < 0.001), whereas no significant changes were observed for SS (*p* = 0.886) and VT (*p* = 0.808). Magnitude analysis indicated substantial reductions in microhardness for FZ and HZ, while SS and VT maintained their mechanical integrity after aging (Fig. 3).

#### Flexural strength and flexural modulus

**Fig. 4:**
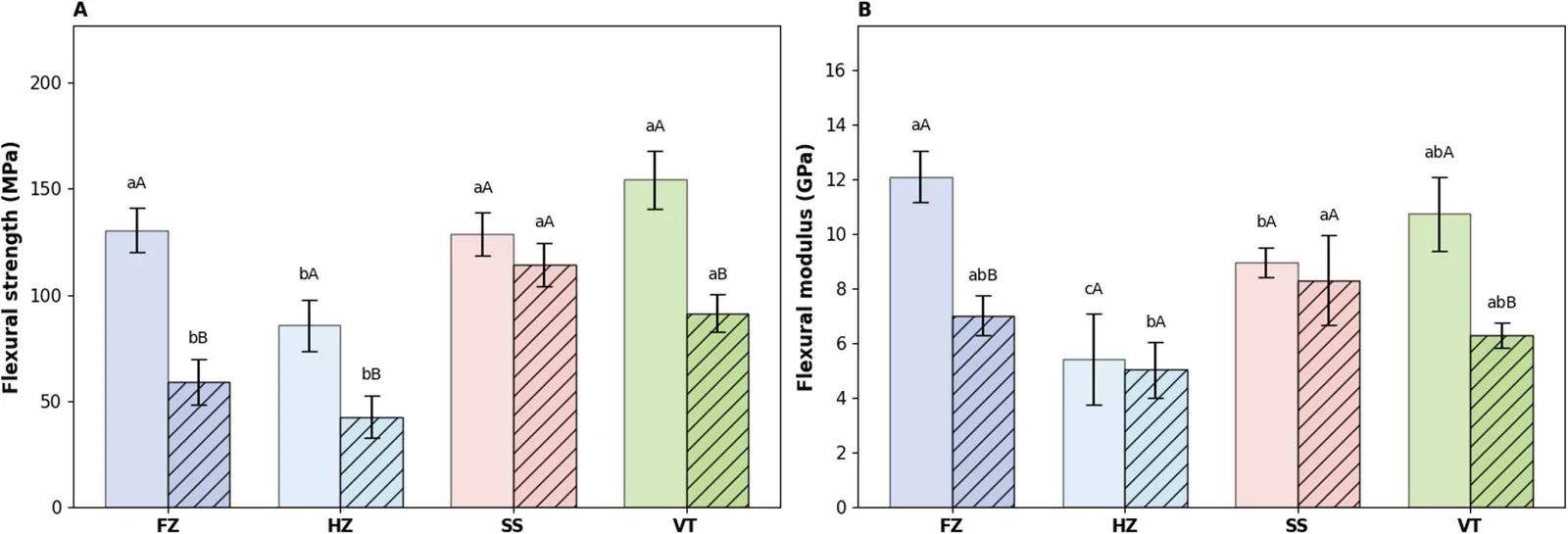
Mean ± standard deviation of flexural strength (MPa) and flexural modulus (GPa) before and after thermocycling. *Different capital letters indicate significant differences for thermocycling (before or after) from Tukey’s test (*p* < 0.05). ** Different lowercase letters indicate significant differences between the materials from Tukey’s test (*p* < 0.05)

The flexural strength (FS) and flexural modulus (FM) results are shown in Fig. 4. Statistical analysis demonstrated that both material type (*p* < 0.001) and thermocycling (*p* < 0.001) significantly influenced FS and FM values. Additionally, a significant interaction between these factors was observed for FS (*p* = 0.005) and FM (*p* < 0.001).

Regarding flexural strength, SS did not exhibit a statistically significant reduction after thermocycling (128.74 ± 27.14 MPa vs. 114.13 ± 29.27 MPa; *p* = 0.215). Before aging, HZ presented the lowest FS values among the evaluated materials (85.74 ± 22.16 MPa; 95% CI: 69.90–101.58), whereas VT showed the highest values (154.29 ± 33.54 MPa; 95% CI: 130.33–178.25).

After thermocycling, a marked reduction in FS was observed for most materials. HZ (42.71 ± 15.09 MPa; 95% CI: 31.92–53.50) and FZ (59.04 ± 16.90 MPa; 95% CI: 46.95–71.13) exhibited the lowest values, while SS maintained the highest flexural strength (114.13 ± 29.27 MPa; 95% CI: 93.19–135.07). VT showed intermediate performance after aging (91.31 ± 8.83 MPa; 95% CI: 85.00–97.62). For flexural modulus, SS and VT demonstrated higher FM values after thermocycling compared to FZ and HZ, indicating better mechanical stability.

#### SEM/EDS

**Fig. 5:**
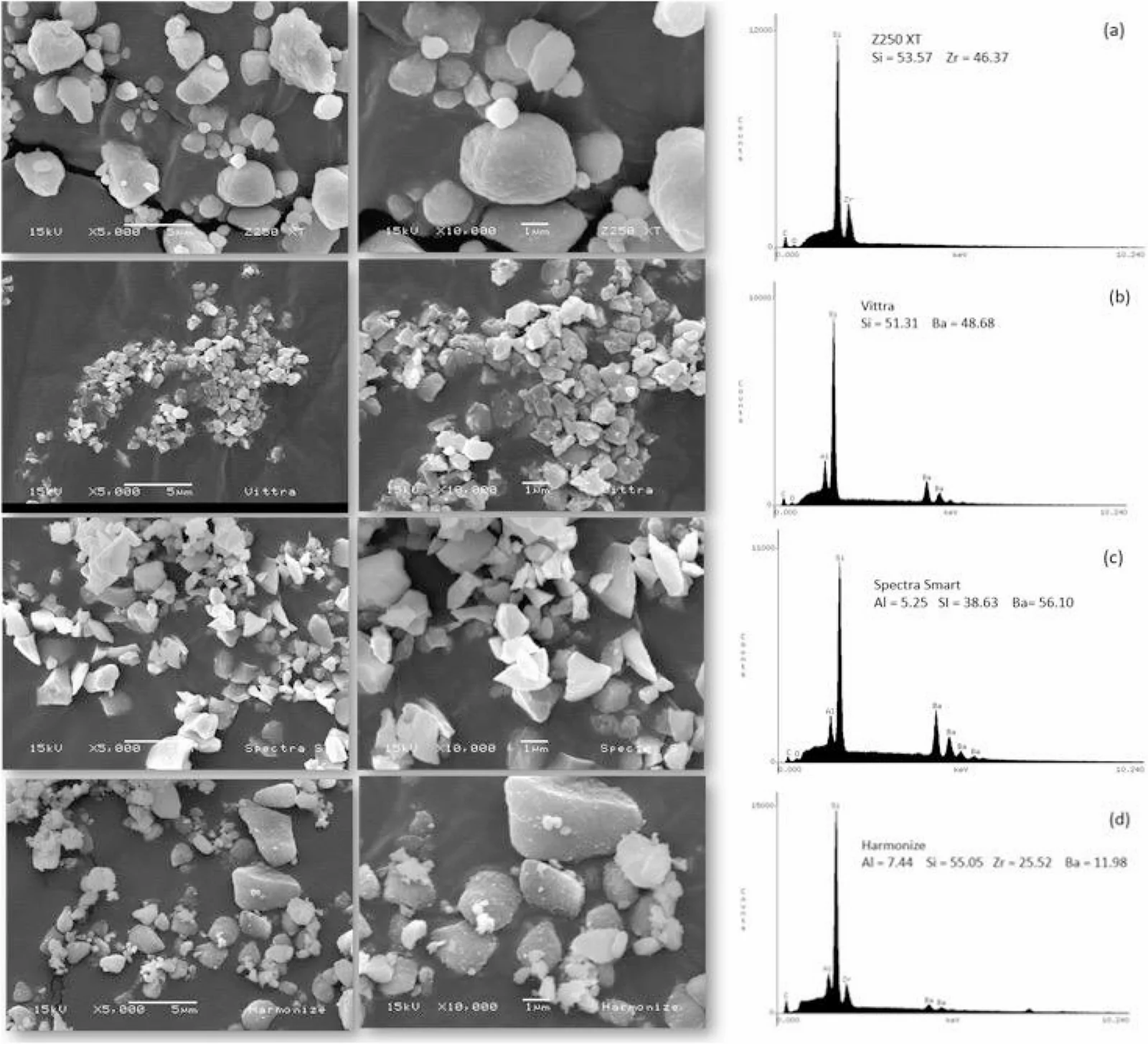
SEM of composites resins particles and graphs concerning the qualitative and semi-quantitative (wt%) EDS analysis of the chemical elements detected in (**a**) Z250 XT, (**b**) Vittra, (**c**) Smart Spectra and (**d**) Harmonize particles

Scanning electron microscopy (SEM) analysis revealed distinct filler morphologies among the composite resins after removal of the organic matrix (Fig. 5). Quantitative particle size analysis was performed using ImageJ software, based on calibrated SEM images. For each material, three representative images (*n* = 3) were analyzed, and at least 30 particles per image were measured (total *n* ≥ 90 particles per material). The FZ resin exhibited two predominant filler morphologies: spherical particles (mean size: ~1.8 ± 1.2 μm) and rhomboid particles (~ 3.5 ± 2.1 μm), with a wide size distribution ranging from approximately 0.1 μm to 8 μm. Energy-dispersive spectroscopy (EDS) identified silicon, oxygen, and zirconium as the main elements.

The VT resin showed irregularly shaped filler particles with a narrower size distribution (mean size: ~0.8 ± 0.3 μm). EDS spectra revealed the presence of barium, aluminum, silicon, and oxygen. The SS resin presented irregular filler particles with a broader size distribution (mean size: ~2.2 ± 1.5 μm), and EDS analysis detected barium, silica, oxygen, and aluminum. The HZ resin exhibited irregular particles (mean size: ~3.0 ± 1.8 μm) along with rhomboid particles (~ 1.5 ± 0.9 μm), with sizes ranging from 0.4 μm to 6 μm. The elemental composition identified by EDS was consistent with silica-based fillers.

## Discussion

The present study investigated the influence of thermocycling on the optical and mechanical behavior of universal resin composites. The results demonstrated that thermocycling significantly affected color stability, whereas mechanical changes were material-dependent. Consequently, both null hypotheses were rejected. Importantly, the magnitude and direction of these changes varied among materials, indicating that performance is governed not only by optical design but also by intrinsic microstructural and chemical characteristics. Although these materials provide simplified shade selection and highly favorable initial blending with tooth structure, the findings indicate that their long-term stability remains vulnerable to hydrolytic and thermal degradation processes inherent to the polymer network, filler-matrix interface, and silane coupling system.

The thermocycling protocol employed in this study (10,000 cycles between 5 and 55 °C) is widely used to simulate thermal stresses occurring in the oral environment. Previous studies have suggested that this number of cycles may correspond approximately to one year of clinical service; however, this equivalence should be interpreted with caution due to the multifactorial nature of intraoral aging conditions [27, 28]. It is important to emphasize that thermocycling primarily reproduces thermal fluctuations and water exposure, but does not fully replicate the complexity of the oral environment, which includes additional factors such as mechanical loading, biofilm activity, enzymatic degradation, and chemical challenges [28].

Color stability is a critical determinant of the long-term success of esthetic restorations, particularly for resin composites designed to rely on optical blending rather than conventional pigmentation [29, 30]. In the present study, thermocycling promoted perceptible color changes in all evaluated materials, as demonstrated by the ΔE₀₀ values. This result corroborates previous studies indicating that aging procedures can compromise the optical performance of resin composites, even those engineered to exploit structural color mechanisms rather than pigment-based color matching [6, 31].

Importantly, when interpreted under clinically established thresholds, all evaluated materials exceeded the acceptability limit for color change. It has been reported that ΔE₀₀ values above approximately 0.8 are perceptible and values above 1.8 are considered clinically unacceptable [32]. Based on this criterion, all composites in the present study demonstrated clinically unacceptable color changes after thermocycling, regardless of the statistical differences observed among them. These findings indicate that none of the materials maintained long-term esthetic stability after aging.

The observed differences among materials may be explained by variations in filler composition, particle size distribution, and resin matrix chemistry. SS presented the lowest ΔE₀₀ values, suggesting superior color stability. SEM analysis revealed that SS and FZ exhibited larger filler particles compared to VT and HZ. Larger filler particles reduce the total resin matrix volume and limit water diffusion pathways, which may mitigate hydrolytic degradation and subsequent changes in refractive index mismatches between the resin matrix and filler particles [33, 34]. This mechanism has been previously described as a key factor influencing optical degradation in resin composites [35].

Conversely, VT showed the highest color variation after thermocycling. The presence of smaller filler particles (0.02–0.7 μm), as reported by the manufacturer and confirmed by SEM, increases the filler–matrix interfacial area. While this feature enhances polishability and initial optical integration, it also increases susceptibility to water sorption and interfacial degradation. Hydrolytic breakdown of silane coupling agents at the filler–matrix interface may lead to microstructural changes that alter light scattering behavior, thereby compromising the structural color effect over time [36, 37].

HZ demonstrated intermediate behavior, with notable color changes following thermocycling. Harmonize relies on a proprietary adaptive response technology that modulates light transmission at different wavelengths to achieve blending. However, this mechanism may be particularly sensitive to changes in refractive index caused by water sorption and thermal stress. The combination of irregular filler morphology and a resin matrix containing Bis-GMA, Bis-EMA, and TEGDMA may further exacerbate this effect, as TEGDMA-rich systems are known to exhibit higher water uptake [38, 39].

Resin composites containing Bis-GMA in their organic matrix tend to exhibit higher translucency when compared with formulations in which this monomer is absent, indicating that the chemical composition of the resin matrix plays a decisive role in the optical behavior of the material [40–41]. In addition to the resin matrix, filler particles also significantly influence light transmission within resin composites, affecting how light is scattered and refracted throughout the material [33]. The blending effect of a restoration is therefore governed by complex optical interactions involving diffuse light scattering and refraction within the composite structure [31]. Incident light is partially reflected by the filler particles, the organic resin matrix, incorporated pigments, and the underlying background, and the combined interaction of these components ultimately determines the perceived color of the restoration [34].

The mechanical properties depend on the composition and the polymerization network created within resin composites during photoactivation [42]. FZ and HZ exhibited a significant reduction in Knoop microhardness after thermocycling, a behavior commonly associated with hydrolytic degradation of the resin matrix and plasticization of the polymer network due to water sorption. Both materials contain relatively hydrophilic monomers, such as Bis-GMA and TEGDMA, which facilitate water diffusion into the composite structure, reducing intermolecular forces and increasing polymer chain mobility, ultimately leading to a decrease in surface hardness after thermal aging [43, 44].

In addition, repeated thermal stresses during thermocycling may induce microcrack formation and filler–matrix interfacial debonding, further contributing to the reduction in resistance to plastic deformation [21, 22]. In contrast, SS and VT exhibited lower initial microhardness values but maintained their Knoop microhardness after thermocycling, as indicated by the absence of statistically significant differences between baseline and aged conditions. Conversely, the pronounced reduction in Knoop microhardness observed for FZ and HZ further supports their increased susceptibility to surface degradation. Since microhardness is directly associated with wear resistance, these changes may lead to faster surface deterioration and loss of anatomical form during clinical service. The stability observed for SS may be attributed to its polymer matrix based on TCD-urethane dimethacrylate, which promotes the formation of a highly cross-linked and more hydrophobic polymer network, significantly reducing water sorption and limiting matrix plasticization during aging [45, 46].

From a magnitude perspective, Knoop microhardness reductions were also substantial, with FZ and HZ exhibiting decreases of approximately 30–45% after thermocycling, while SS and VT showed minimal changes (< 10%). These reductions are clinically relevant, as microhardness is directly associated with wear resistance and surface integrity. A decrease of this magnitude may lead to accelerated surface degradation, increased roughness, and higher susceptibility to staining and biofilm accumulation over time.

The mechanical performance of resin composites was further assessed by the three-point bending test, which remains a gold-standard method for evaluating flexural strength and elastic modulus and is recommended by ISO standards for dental restorative materials [47]. According to ISO requirements, a minimum flexural strength of 80 MPa is considered acceptable for resin composites intended for clinical use [48]. At baseline, FZ and SS exhibited the highest flexural strength values, exceeding the ISO threshold, whereas HZ and VT showed lower values, although still within clinically acceptable ranges. These findings highlight that, despite their simplified shade-matching concept, universal and single-shade composites can present mechanical performance comparable to conventional nanohybrid materials [49].

Following thermocycling, a significant reduction in flexural strength was observed for FZ, HZ, and VT; however, VT maintained flexural strength values above the minimum threshold of 80 MPa recommended by ISO standards, whereas SS exhibited statistically similar values before and after aging. From a magnitude-based perspective, the reduction in flexural strength was substantial, exceeding 50% for FZ and HZ and approximately 40% for VT, while SS showed only a minimal reduction (~ 10%). These differences indicate markedly distinct degradation patterns among the evaluated materials. Importantly, FZ and HZ exhibited flexural strength values below the ISO 4049 minimum threshold (80 MPa) after thermocycling [47]. This finding represents a critical mechanical failure under simulated aging conditions and cannot be overlooked when considering clinical applicability. Materials that fail to meet ISO requirements after aging may present compromised structural integrity, increasing the risk of restoration failure under functional loading. From a clinical perspective, these materials may therefore be less suitable for posterior restorations or stress-bearing areas, where higher occlusal forces are expected, as flexural strength has been shown to be closely associated with the ability of resin composites to withstand masticatory stresses and long-term functional wear [24, 48].

Since flexural strength of resin composites has been correlated with clinical wear behavior rather than fracture resistance, FZ and HZ may be more susceptible to increased clinical wear when compared with the other materials evaluated in this study [24]. This behavior may be partly explained by the presence of Bis-GMA in the resin matrices of both FZ and HZ, as this monomer is associated with higher water sorption and plasticization due to its hydroxyl groups, which facilitate hydrolytic degradation and weaken the polymer network under aging conditions [50]. In agreement with the present findings, a previous study evaluating universal resin composites after thermocycling reported a substantial reduction in flexural strength, with materials containing similar filler content exhibiting decreases exceeding 50% of their baseline values, reinforcing the susceptibility of Bis-GMA–based systems to thermal aging [49].

The superior stability observed for SS may be explained by its unique polymer chemistry, which includes TCD-urethane dimethacrylate. This monomer promotes the formation of a highly cross-linked and rigid polymer network with reduced hydrophilicity, limiting water sorption and enhancing resistance to aging-induced degradation [51, 52]. Flexural modulus followed a similar trend, with FZ and SS presenting higher baseline values, while HZ exhibited the lowest elastic modulus. Since elastic modulus is strongly correlated with filler content, filler morphology, and distribution, these differences can be attributed to variations in inorganic loading and particle architecture among the materials. Although VT showed a relatively high baseline modulus, thermocycling significantly reduced its elastic modulus, suggesting that internal structural degradation occurred despite the preservation of surface microhardness. This reinforces the concept that surface properties and bulk mechanical behavior may respond differently to aging challenges.

The favorable mechanical performance observed for SS may be explained by its resin matrix based on TCD-urethaneacrylate. According to Durner et al., [45] TCD-based monomers exhibit high reactivity and allow the formation of a highly cross-linked polymer network with elevated degree of conversion, even without the use of low-molecular-weight diluent monomers. In addition, the bulky molecular structure and lower intrinsic viscosity of TCD-urethaneacrylate contribute to reduced water sorption and limited polymer chain mobility when compared with conventional Bis-GMA-based systems. These characteristics result in improved chemical stability and resistance to mechanical degradation, which supports the maintenance of mechanical properties observed for SS in the present study.

Collectively, these findings indicate that the optical blending behavior of universal resin composites is not inherently stable over time and may deteriorate after aging. From a clinical perspective, this limitation is particularly relevant, as restorations initially exhibiting excellent optical blending may develop perceptible and clinically unacceptable color mismatch over time. In addition, the mechanical degradation observed, particularly the substantial reductions in flexural strength and microhardness for certain materials, further compromises their long-term performance. Materials that exhibited reductions exceeding 50% in flexural strength and 30–45% in microhardness, as well as values below the ISO 4049 threshold after aging, may present reduced resistance to functional loading, increased wear, and a higher susceptibility to structural failure. Overall, both esthetic and mechanical stability appear to be strongly material-dependent, and the long-term clinical reliability of single-shade composites may be limited, especially in stress-bearing or highly esthetic regions.

The authors also recognize, as reported by previous studies, that investigations on single-shade resin composites present inherent limitations. Thermocycling is a well-established method for simulating thermal stress and water diffusion in the oral environment. However, it does not fully reproduce the complexity of intraoral conditions, where mechanical loading, chemical challenges, and biofilm activity interact simultaneously. Therefore, the aging protocol used in this study should be interpreted as a controlled and reproducible model, which allows standardized comparison between materials, rather than a direct representation of clinical reality.

The solvent-based protocol used for organic matrix removal prior to SEM analysis also presents inherent limitations. Although sequential washing was performed to enhance matrix dissolution, complete elimination of the organic phase cannot be fully guaranteed without additional analytical confirmation. Consequently, minor residual components may have influenced the morphological assessment. Additionally, although filler loading plays a critical role in the mechanical behavior of resin composites, complete volumetric data were not available for all materials, limiting direct quantitative correlations. Nevertheless, the combined evaluation of optical, mechanical, and microstructural properties provides a comprehensive and clinically relevant understanding of material performance.

Finally, while in vitro findings cannot be directly extrapolated to long-term clinical outcomes, the present study provides relevant insights into the behavior of universal and single-shade resin composites under controlled aging conditions, supporting future investigations and clinical interpretation.

## Conclusions

Within the limitations of the present study, it was concluded that both null hypotheses were rejected:

- Thermocycling induced clinically relevant color alterations in all evaluated universal resin composites, indicating instability of their single-shade behavior after aging. All materials exceeded perceptibility and acceptability thresholds, indicating loss of the optical blending effect after aging.
- Thermocycling affected the mechanical properties of the evaluated universal resin composites in a material-dependent manner. Significant reductions in flexural strength and microhardness indicate compromised mechanical stability after aging.

## Data Availability

The datasets used and/or analysed during the current study are available from the corresponding author on reasonable request.
